# Filamentation Is Associated with Reduced Pathogenicity of Multiple Non-*albicans Candida* Species

**DOI:** 10.1128/mSphere.00656-19

**Published:** 2019-10-16

**Authors:** Mohua Banerjee, Anna L. Lazzell, Jesus A. Romo, Jose L. Lopez-Ribot, David Kadosh

**Affiliations:** a Department of Microbiology, Immunology and Molecular Genetics, University of Texas Health Science Center at San Antonio, San Antonio, Texas, USA; b Department of Biology and South Texas Center for Emerging Infectious Diseases, The University of Texas at San Antonio, San Antonio, Texas, USA; Carnegie Mellon University

**Keywords:** candidiasis, infectious disease, mycology, morphology, pathogenicity, *Candida* species, evolution, filamentation, gene expression

## Abstract

Candidiasis affects a wide variety of immunocompromised and medically compromised patients. Candida albicans, a major human fungal pathogen, accounts for about 50% of all cases, while the remainder are caused by the less pathogenic non-*albicans Candida* species (NACS). These species are believed to be less pathogenic, in part, because they do not filament as readily or robustly as C. albicans, although definitive evidence is lacking. To address this question, we used strains for two NACS, Candida tropicalis and Candida parapsilosis, which were genetically engineered to constitutively express the key transcriptional regulator *UME6* and drive strong filamentation both *in vitro* and during infection *in vivo*. Unexpectedly, both strains showed a dramatic reduction in organ fungal burden in response to *UME6* expression. Consistent with these findings, we observed that a C. tropicalis hyperfilamentous mutant was significantly reduced and a filamentation-defective mutant was slightly increased for organ fungal burden. Comprehensive immune profiling generally did not reveal any significant changes in the host response to *UME6* expression in the NACS that could explain the increased clearance of infection. Interestingly, whole-genome transcriptional profiling indicated that while genes important for filamentation were induced by *UME6* expression in C. tropicalis and C. parapsilosis, other genes involved in a variety of processes important for pathogenesis were strongly downregulated. These findings suggest that there are fundamental evolutionary differences in the relationship between morphology and pathogenicity among *Candida* species and that NACS do not necessarily possess the same virulence properties as C. albicans.

**IMPORTANCE** Many immunocompromised individuals, including HIV/AIDS and cancer patients, are susceptible to candidiasis. About half of all cases are caused by the major fungal pathogen Candida albicans, whereas the remainder are due to less pathogenic non-*albicans Candida* species (NACS). Generation of filamentous cells represents a major virulence property of C. albicans, and the NACS are believed to be less pathogenic, in part, because they do not filament as well as C. albicans does. To address this question, we determined the pathogenicity of two NACS strains that have been genetically engineered to promote filamentation during infection. Surprisingly, these strains showed a dramatic reduction in pathogenicity. The host immune response did not appear to be affected. However, unlike C. albicans, filamentation of the NACS was associated with downregulation of several genes important for pathogenicity processes. Our results suggest that there are fundamental evolutionary differences in the relationship between filamentation and pathogenesis in NACS compared to C. albicans.

## Abstract (precis)

Many immunocompromised individuals, including HIV/AIDS and cancer patients, are susceptible to candidiasis. About half of all cases are caused by the major fungal pathogen Candida albicans, whereas the remainder are due to less pathogenic non-*albicans Candida* species (NACS). Generation of filamentous cells represents a major virulence property of C. albicans, and the NACS are believed to be less pathogenic, in part, because they do not filament as well as C. albicans does. To address this question, we determined the pathogenicity of two NACS strains that have been genetically engineered to promote filamentation during infection. Surprisingly, these strains showed a dramatic reduction in pathogenicity. The host immune response did not appear to be affected. However, unlike C. albicans, filamentation of the NACS was associated with downregulation of several genes important for pathogenicity processes. Our results suggest that there are fundamental evolutionary differences in the relationship between filamentation and pathogenesis in NACS compared to C. albicans.

## OBSERVATION

*Candida* species account for the large majority of human fungal infections. These species can cause both mucosal infections, such as oral and vaginal thrush, and more serious life-threatening systemic bloodstream infections (1–3). Individuals with a compromised immune system, including cancer patients on chemotherapy, organ transplant recipients, and HIV/AIDS patients, are highly susceptible to infection (2, 3). Approximately 50% of all *Candida* bloodstream infections in the United States can be attributed to Candida albicans, while the remainder are due to a variety of inherently less pathogenic non-*albicans Candida* species (NACS) (4). Infections by NACS are on the rise, and several of these species show increased resistance to commonly used antifungal therapies (5).

C. albicans possesses a variety of virulence traits, including the ability to undergo a reversible morphological transition from single budding yeast cells to filaments, which are best described as elongated cells attached end to end (6). Multiple independent lines of evidence have suggested a strong association between the C. albicans yeast-filament transition and virulence (7–11), including our previous demonstration that constitutive high-level expression of the filament-specific transcriptional regulator *UME6* promotes this morphological transition and enhances C. albicans virulence, as well as tissue invasion, in a mouse model of systemic candidiasis (12).

Considerably less research has focused on NACS. These species are thought to be less pathogenic than C. albicans for a variety of reasons, including a reduced ability to adhere to host cells, secrete degradative enzymes, and form biofilms (13, 14). They are also generally more sensitive to cell stresses encountered in the host environment and do not filament as readily or robustly as C. albicans (14). We have previously shown that certain C. albicans morphological regulatory functions are evolutionarily conserved in several NACS, including Candida tropicalis and Candida parapsilosis (15). As in C. albicans, orthologs of *UME6* are transcriptionally induced in these species during filamentation, although at a reduced level and, in the case of C. parapsilosis, with delayed timing. In addition, as is the case for C. albicans, constitutive high-level expression of *UME6* orthologs is sufficient to promote strong filamentation in C. tropicalis and C. parapsilosis; orthologs of several, but not all, C. albicans filament-specific genes were also induced in response to *UME6* expression in these species (15).

**FIG 1:**
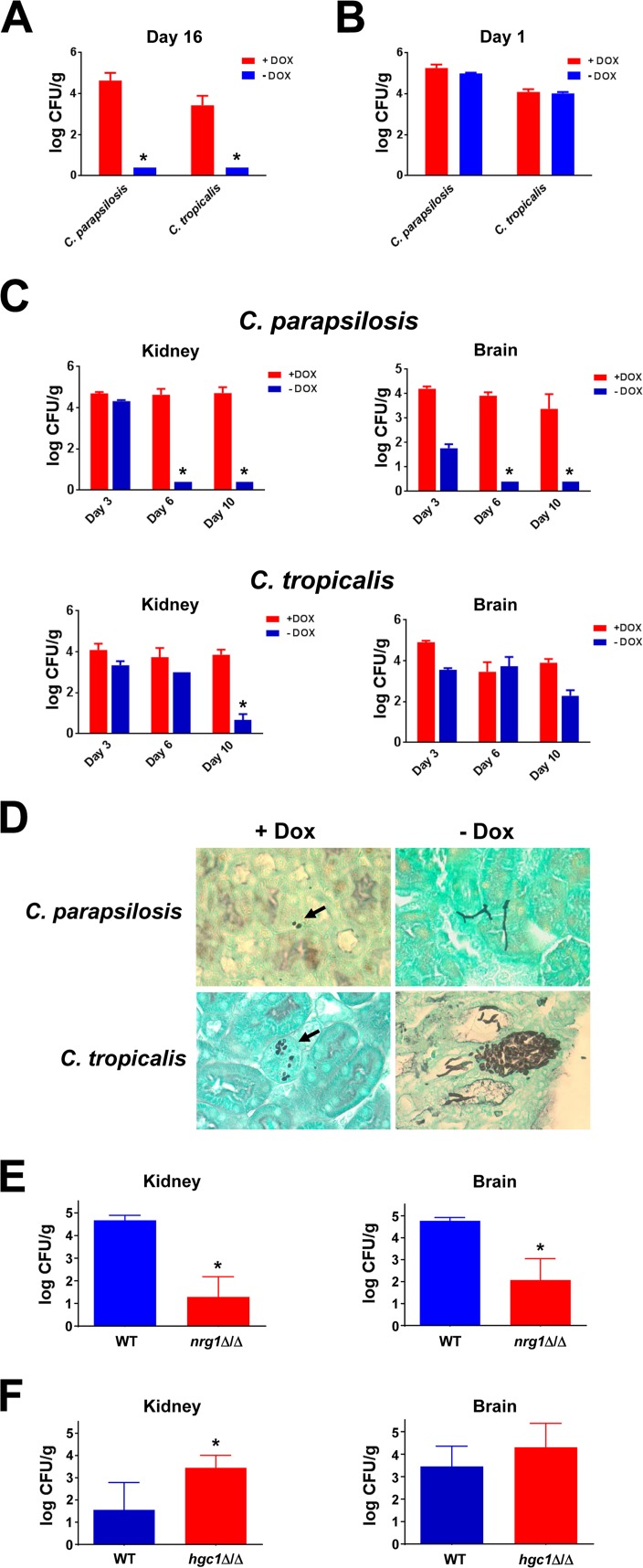
Filamentation is associated with reduced fungal burden of C. tropicalis and C. parapsilosis in a mouse model of systemic candidiasis. (A) C. tropicalis (4 × 10^4^ CFU) and C. parapsilosis (4 × 10^6^ CFU) *tetO-UME6* strains were used to inoculate female BALB/c mice (6 to 8 weeks old) by tail vein injection. Half the mice were placed on drinking water with 2 mg/ml Dox (*n* = 5 mice/group). All mice were sacrificed at 16 days postinfection, kidneys were harvested, and fungal burdens were determined. For both species, the reduction in fungal burden in the −Dox versus +Dox groups was statistically significant (*, *P* < 0.01) as determined by a Mann-Whitney test. (B) The experiment in panel A was repeated using 2.1 × 10^5^ CFU of C. tropicalis and 4.2 × 10^6^ CFU of C. parapsilosis *tetO-UME6* strains, and all mice were sacrificed at 24 h postinfection for kidney fungal burden determination. (C) The experiment in panel A was repeated for the C. parapsilosis and C. tropicalis *tetO-UME6* strains using inoculum sizes of 4.5 × 10^6^ CFU and 3.0 × 10^4^ CFU, respectively. Mice were sacrificed at the indicated postinfection time points, and fungal burdens were determined for the indicated organs (*, *P* < 0.05, using a Mann-Whitney test). Please note that the fungal burden value for kidneys infected with the C. tropicalis *tetO-UME6* strain in the absence of Dox at day 6 represents the lower limit of detection. (D) Examples showing the effect of *UME6* expression on C. tropicalis and C. parapsilosis morphology during infection *in vivo*. Kidneys from mice infected with *tetO-UME6* strains from the indicated species were harvested, fixed, embedded in paraffin, stained with Grocott-Gomori methenamine silver (GMS), and visualized by light microscopy (fungal cells shown in black). Black arrows indicate yeast cells. (E) The C. tropicalis WT (3.7 × 10^5^ CFU) and *nrg1*Δ/Δ (3.5 × 10^5^ CFU) strains were used to inoculate female BALB/c mice (6 to 8 weeks old) by tail vein injection (*n* = 5). All mice were sacrificed at 6 days postinfection, kidneys were harvested, and fungal burdens were determined. The reduction in fungal burdens in mice infected with *nrg1*Δ/Δ versus WT strains was statistically significant (*, *P* < 0.01) as determined by a Mann-Whitney test. (F) The experiment in panel E was repeated using 2.9 × 10^4^ and 3.1 × 10^4^ CFU of C. tropicalis WT and *hgc1*Δ/Δ strains, respectively. The increase in kidney fungal burden in mice infected with *hgc1*Δ/Δ versus WT strains was statistically significant (*, *P* < 0.05) as determined by a Mann-Whitney test.

In order to determine the specific effect of filamentation on the pathogenicity of C. parapsilosis and C. tropicalis during infection *in vivo*, we used our previously constructed *tetO-CtUME6* and *tetO-CpUME6* strains (15). In the absence of doxycycline (Dox), a repressor of the tetracycline operator (*tetO*), one allele of *UME6* in these strains is expressed at constitutive high levels, generating a highly filamentous morphology, whereas in the presence of Dox this allele is shut off and cells grow as yeast. Both C. tropicalis and C. parapsilosis *tetO-UME6* strains were used to inoculate female BALB/c mice by tail vein injection. Half the mice in each group were supplied with drinking water containing Dox. At day 16 postinfection, all mice were sacrificed and kidneys were harvested for fungal burden analysis. Unexpectedly, as shown in Fig. 1A, there was a significant reduction in fungal burden in the −Dox versus +Dox group for both species. Indeed, fungal burdens in the −Dox groups were below the limit of detection, suggesting that the infections had cleared. In a similar experiment, mice sacrificed at 24 h postinfection showed equivalent kidney (Fig. 1B) and brain (data not shown) fungal burdens in both +Dox and −Dox groups for each species. In order to determine the time course for organ clearance and whether clearance occurred in multiple organs, we next performed a timed-sacrifice experiment using both C. parapsilosis and C. tropicalis *tetO-UME6* strains and examined fungal burdens in both kidneys and brains at different days postinfection (Fig. 1C). Interestingly, at the day 3 postinfection time point the +Dox and −Dox groups inoculated with the C. parapsilosis *tetO-UME6* strain showed roughly equivalent kidney fungal burdens, although fungal burden was partly reduced in the brains of −Dox versus +Dox animals. At day 6 and day 10, there was a dramatic reduction in both kidney and brain fungal burden in the −Dox versus +Dox groups. As in the previous experiment, fungal burdens for the −Dox groups at these later time points were below the limit of detection. A similar trend in results, though less pronounced, was observed for the C. tropicalis *tetO-UME6* strain (Fig. 1C). Interestingly, these results suggested that constitutive high-level *UME6* expression is sufficient to significantly reduce both C. parapsilosis and C. tropicalis fungal burden, eventually leading to clearance of the infection from multiple organs. Histological analysis of infected kidneys was performed to confirm that *UME6* expression was able to promote filamentation during infection *in vivo*. As shown in Fig. 1D, both strains were observed to grow primarily in the yeast form in infected kidneys from the +Dox group. In contrast, in the absence of Dox the C. tropicalis *tetO-UME6* strain was found as a mixture of yeast and filaments and the C. parapsilosis *tetO-UME6* strain grew primarily as filaments (Fig. 1D). In order to determine whether organ clearance was more generally associated with morphology, mice were inoculated with either a hyperfilamentous C. tropicalis *nrg1*Δ/Δ mutant (15) or a filamentation-defective C. tropicalis *hgc1*Δ/Δ strain (16). Consistent with our previous results, the *nrg1*Δ/Δ mutant showed significantly reduced fungal burden compared to the wild-type (WT) strain in both the kidneys and brain (Fig. 1E). Also, in contrast to most filamentation-defective mutants of C. albicans, the C. tropicalis *hgc1*Δ/Δ mutant showed slightly increased organ fungal burdens compared to those of the WT control (Fig. 1F).

We next sought to determine whether organ clearance that is observed in response to *UME6* expression in C. tropicalis and C. parapsilosis occurs as the result of an altered host response. Kidney homogenates were prepared from mice placed on drinking water in the presence or absence of Dox, inoculated with C. tropicalis and C. parapsilosis *tetO-UME6* strains, and sacrificed at day 1 postinfection when fungal burdens are equivalent in +Dox and −Dox groups. These homogenates were then used to carry out comprehensive multianalyte profiling of over 50 different cytokines, chemokines, and other host markers of infection. In general, we did not detect significant differences in the levels of these analytes when comparing −Dox with +Dox groups, which could indicate an altered immune response (see Fig. S1 in the supplemental material). However, we did observe a significant reduction in both the C. tropicalis and C. parapsilosis −Dox versus +Dox ratio for myoglobin. Given that myoglobin levels are known to be correlated with tissue damage (17), these results are consistent with our previous findings and suggest that greater tissue damage occurs in response to C. tropicalis and C. parapsilosis yeast than to filamentous cells.

10.1128/mSphere.00656-19.2 FIG S1 *UME6* expression does not generally cause significant changes in the levels of cytokines, chemokines, and other host immune markers during a systemic infection of C. tropicalis or C. parapsilosis. Pooled kidney homogenates (*n* = 5) were obtained from mice placed on drinking water in the presence or absence of 2 mg/ml Dox, infected with *tetO*-*CtUME6* and *tetO*-*CpUME6* strains, and sacrificed at 1 day postinfection. Multiplex analysis using mouse multianalyte profiling (MAP; Rules Based Medicine) was carried out to measure the levels of a variety of different host analytes and immune markers. Comparative ratios in the absence versus presence of Dox are shown for each analyte/immune marker in C. parapsilosis (A) and C. tropicalis (B). The solid line indicates a ratio of 1, and dotted lines indicate ratios of 0.5 and 2. Analytes/immune markers showing ratios above or below the dotted lines were considered to be significantly affected by *UME6* expression. Download FIG S1, PDF file, 0.01 MB. Copyright © 2019 Banerjee et al. 2019 Banerjee et al. This content is distributed under the terms of the Creative Commons Attribution 4.0 International license.

**FIG 2:**
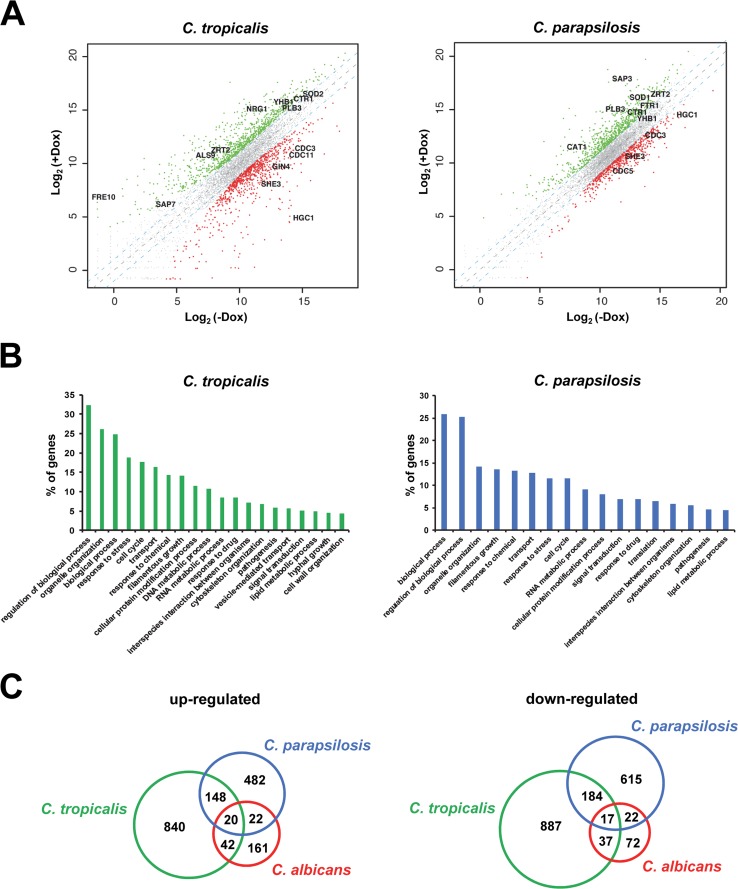
Transcriptional profile of C. tropicalis and C. parapsilosis in response to *UME6* expression. (A) Scatter plots showing gene expression changes for C. tropicalis and C. parapsilosis *tetO-UME6* strains grown in the absence versus presence of doxycycline (Dox). Axes represent log_2_(averaged normalized read counts) for +Dox and −Dox. Transcripts that are differentially expressed ≥2-fold (dashed lines) are indicated in red (upregulated) and green (downregulated). Transcripts of interest are labeled in black. (B) GO slim mapper (www.candidagenome.org) analysis showing percent representation of process gene classes in the sets of genes induced ≥2-fold in response to *UME6* expression in C. tropicalis and C. parapsilosis. Gene classes showing less than 4% representation are not shown. (C) Venn diagrams showing overlap of gene orthologs that are upregulated or downregulated in response to *UME6* expression in C. tropicalis, C. parapsilosis, and C. albicans. C. albicans genes showing differential expression in response to *UME6* induction have been described previously (18).

We were also interested in gaining a better understanding of the basis for organ clearance in response to *UME6* expression in C. tropicalis and C. parapsilosis. Thus, we performed whole-genome transcriptional profiling experiments. Both *tetO-CtUME6* and *tetO-CpUME6* strains were grown in the presence and absence of Dox to generate yeast and filaments (Fig. S2), respectively, and cells were harvested for RNA preparation and transcriptome sequencing (RNA-seq) analysis. As indicated in Fig. 2A as well as Tables S1 and S2, we observed that large sets of genes showed significantly increased and decreased expression in response to *UME6* induction in both strains. In contrast, as expected, very few genes showed significant expression changes in the absence versus presence of Dox for both C. tropicalis and C. parapsilosis WT control strains. A gene ontology (GO) analysis identified several common gene classes that were induced in response to *UME6* expression in both C. tropicalis and C. parapsilosis (Fig. 2B and Data Sets S1 and S3). In addition to filamentous growth, as expected, these gene classes included organelle organization, stress response, cell cycle, and transport. In C. parapsilosis, genes involved in amino acid metabolism were overrepresented compared to the genome as a whole, whereas genes associated with mitosis and the cell cycle were overrepresented in C. tropicalis. Among downregulated genes, those involved in carbohydrate metabolism were overrepresented in both C. tropicalis and C. parapsilosis, whereas genes associated with lipid catabolic processes were strongly overrepresented only in C. parapsilosis (Data Sets S2 and S4).

10.1128/mSphere.00656-19.3 FIG S2 Validation that *UME6* expression drives filamentation in cells of *tetO*-*CpUME6* and *tetO*-*CtUME6* strains used for transcriptional profiling. The indicated strains were grown at 30°C in synthetic complete (SC) medium (C. parapsilosis) or yeast extract-peptone-dextrose (YEPD) plus 50% fetal bovine serum (FBS) (C. tropicalis) in the presence or absence of 100 ng/ml doxycycline (Dox) for RNA preparation as described in Text S1. At 36 and 24 hours, aliquots of cells from C. parapsilosis (A) and C. tropicalis (B) strains, respectively, were fixed in 4.5% formaldehyde, washed in 1× phosphate-buffered saline (PBS) twice, and visualized by differential inference contrast (DIC) microscopy. Bar, 10 μm. *Cp*, C. parapsilosis; *Ct*, C. tropicalis. Download FIG S2, PDF file, 0.6 MB. Copyright © 2019 Banerjee et al. 2019 Banerjee et al. This content is distributed under the terms of the Creative Commons Attribution 4.0 International license.

10.1128/mSphere.00656-19.1 TEXT S1 Supplemental materials and methods. Download Text S1, DOCX file, 0.04 MB. Copyright © 2019 Banerjee et al. 2019 Banerjee et al. This content is distributed under the terms of the Creative Commons Attribution 4.0 International license.

10.1128/mSphere.00656-19.4 TABLE S1 Number of genes showing altered expression in the absence versus presence of doxycycline in Candida parapsilosis *tetO-CpUME6* and wild-type strains. Download Table S1, DOCX file, 0.02 MB. Copyright © 2019 Banerjee et al. 2019 Banerjee et al. This content is distributed under the terms of the Creative Commons Attribution 4.0 International license.

10.1128/mSphere.00656-19.5 TABLE S2 Number of genes showing altered expression in the absence versus presence of doxycycline in Candida tropicalis *tetO-CtUME6* and wild-type strains. Download Table S2, DOCX file, 0.02 MB. Copyright © 2019 Banerjee et al. 2019 Banerjee et al. This content is distributed under the terms of the Creative Commons Attribution 4.0 International license.

Orthologs of several genes known to be induced in response to C. albicans *UME6* expression (18) were also induced in both C. tropicalis and C. parapsilosis. As an independent confirmation of our RNA-seq results, we have previously demonstrated by Northern analysis that a few of these genes (e.g., *UME6*, *HGC1*, and *PHR1*) are induced in both NACS (15). In addition to *HGC1*, which encodes a cyclin-like protein important for septin phosphorylation (19), a number of other genes that play direct roles in the filamentation process, including the *CDC3* septin and *SHE3* mRNA-binding protein, were induced in all three *Candida* species (Fig. 2A and Data Sets S1 and S3); the *CDC11* septin, previously shown to be induced by *UME6* in C. albicans (18), was also induced in C. tropicalis. Interestingly, however, orthologs of several C. albicans genes involved in processes important for pathogenesis were significantly downregulated in response to *UME6* expression in both C. tropicalis and C. parapsilosis but not C. albicans (Fig. 2A and Data Sets S2 and S4) (18). These genes included *SOD2*, a superoxide dismutase important for combating oxidative stress, and *YHB1*, important for adapting to nitrosative stress, as well as a variety of genes important for iron, zinc, and copper transport/regulation (e.g., *CTR1*, *ZRT1*, *ZRT2*, and *FRE9*). Multiple members of the secreted aspartyl protease (*SAP*) gene family as well as the *PLB3* phospholipase, which is likely important for host cell degradation, were also downregulated in response to *UME6* expression in both C. tropicalis and C. parapsilosis. As in C. albicans (18), *CtNRG1*, previously shown to function as a repressor of filamentation in C. tropicalis (15), was downregulated in response to *CtUME6* expression, which is again consistent with the notion that mechanisms important for induction of filamentation by *UME6* are conserved among *Candida* species. A three-way comparison of gene orthologs regulated by *UME6* in C. tropicalis, C. parapsilosis, and C. albicans (Fig. 2C) revealed that orthologs of about one-third of C. albicans genes are upregulated in C. tropicalis and/or C. parapsilosis whereas orthologs of over one-half of C. albicans genes downregulated by *UME6* were also downregulated in C. tropicalis and C. parapsilosis. In the future, it will be useful to determine whether similar gene expression patterns are observed in response to expression of other activators of filamentation in NACS.

In this study, we have used a variety of approaches to more specifically define the relationship between morphology and pathogenicity in two NACS, C. tropicalis and C. parapsilosis. Surprisingly, unlike the situation in C. albicans, we find that under our experimental conditions the transition from yeast cells to filaments is associated with reduced pathogenicity as well as reduced expression of certain genes involved in pathogenicity-related processes. At this point, however, it is unclear whether the clearance effect is due to filamentation *per se* or some other process associated with filamentation (e.g., lowered expression of pathogenicity genes). With respect to C. parapsilosis, our results are consistent with those of a previous study demonstrating that the ability of this species to invade the oral epithelium does not correlate with formation of pseudohyphae (20). Also in support of our findings, a different study has found that a hyperfilamentous C. parapsilosis mutant is significantly attenuated for pathogenicity in both a *Galleria* model and a mouse model of candidiasis (21). While previous reports have suggested a correlation between C. tropicalis filamentation ability and the ability to invade/damage epithelial cells, it is important to note that these experiments were carried out using *in vitro* systems rather than animal models (22, 23). A previous study showing this correlation during infection used immunosuppressed animals and a significantly larger inoculum size (24). We have also observed that increasing inoculum size for the *tetO-CtUME6* strain reduces the organ clearance effect (our unpublished results). An independent previous study has shown, by histological analysis, that C. tropicalis cells injected at smaller inoculum sizes equivalent to those used in our experiments typically grow in the yeast form, whereas greater inoculum sizes of C. tropicalis are correlated with an increased proportion of filamentous cells in kidneys (25). Interestingly, our results suggest that forcing filamentation of C. tropicalis at smaller inoculum sizes, at which cells would otherwise grow in the yeast form during infection *in vivo*, leads to organ clearance. Importantly, these findings suggest not only that filamentation can confer an evolutionary disadvantage for C. tropicalis and C. parapsilosis during infection but also that there are fundamental evolutionary differences in the relationship between morphology and pathogenicity among *Candida* species. More specifically, in NACS other processes that are independent of the morphological transition are likely to play a more prominent role in pathogenesis. On a broader level, our findings are significant because they suggest that not everything we learn about pathogenicity and virulence-related processes in C. albicans can be directly applied to NACS, but rather the virulence traits of these species need to be studied in their own right.

For a detailed description of experimental procedures used in this study, please see Text S1. All research using animals was approved by the Institutional Animal Care and Use Committee at The University of Texas at San Antonio.

## 

### Data availability.

Raw RNA sequencing data for this study have been deposited at the NCBI Gene Expression Omnibus (GEO) database (accession number GSE134321).

10.1128/mSphere.00656-19.6 TABLE S3 Primers used in this study. Download Table S3, DOCX file, 0.01 MB. Copyright © 2019 Banerjee et al. 2019 Banerjee et al. This content is distributed under the terms of the Creative Commons Attribution 4.0 International license.

10.1128/mSphere.00656-19.7 DATA SET S1 C. tropicalis genes induced ≥2-fold in response to *CtUME6* expression and corresponding Gene Ontology (GO) analysis. Download Data Set S1, XLSX file, 0.3 MB. Copyright © 2019 Banerjee et al. 2019 Banerjee et al. This content is distributed under the terms of the Creative Commons Attribution 4.0 International license.

10.1128/mSphere.00656-19.8 DATA SET S2 C. tropicalis genes down-regulated ≥2-fold in response to *CtUME6* expression and corresponding Gene Ontology (GO) analysis. Download Data Set S2, XLSX file, 0.2 MB. Copyright © 2019 Banerjee et al. 2019 Banerjee et al. This content is distributed under the terms of the Creative Commons Attribution 4.0 International license.

10.1128/mSphere.00656-19.9 DATA SET S3 C. parapsilosis genes induced ≥2-fold in response to *CpUME6* expression and corresponding Gene Ontology (GO) analysis. Download Data Set S3, XLSX file, 0.1 MB. Copyright © 2019 Banerjee et al. 2019 Banerjee et al. This content is distributed under the terms of the Creative Commons Attribution 4.0 International license.

10.1128/mSphere.00656-19.10 DATA SET S4 C. parapsilosis genes down-regulated ≥2-fold in response to *CpUME6* expression and corresponding Gene Ontology (GO) analysis. Download Data Set S4, XLSX file, 0.2 MB. Copyright © 2019 Banerjee et al. 2019 Banerjee et al. This content is distributed under the terms of the Creative Commons Attribution 4.0 International license.

## References

[1] Odds FC 1988 *Candida* and candidosis, 2nd ed Baillière Tindall, London, United Kingdom.

[2] Dupont PF 1995 *Candida albicans*, the opportunist. A cellular and molecular perspective. J Am Podiatr Med Assoc 85:104–115. doi:10.7547/87507315-85-2-104. 7877106

[3] Weig M, Gross U, Muhlschlegel F 1998 Clinical aspects and pathogenesis of *Candida* infection. Trends Microbiol 6:468–470. doi:10.1016/s0966-842x(98)01407-3. 10036723

[4] McCarty TP, Pappas PG 2016 Invasive candidiasis. Infect Dis Clin North Am 30:103–124. doi:10.1016/j.idc.2015.10.013. 26739610

[5] Patel PK, Erlandsen JE, Kirkpatrick WR, Berg DK, Westbrook SD, Louden C, Cornell JE, Thompson GR, Vallor AC, Wickes BL, Wiederhold NP, Redding SW, Patterson TF 2012 The changing epidemiology of oropharyngeal candidiasis in patients with HIV/AIDS in the era of antiretroviral therapy. AIDS Res Treat 2012:262471. doi:10.1155/2012/262471. 22970352 PMC3434376

[6] Sudbery P, Gow N, Berman J 2004 The distinct morphogenic states of *Candida albicans*. Trends Microbiol 12:317–324. doi:10.1016/j.tim.2004.05.008. 15223059

[7] Lo HJ, Kohler JR, DiDomenico B, Loebenberg D, Cacciapuoti A, Fink GR 1997 Nonfilamentous *C. albicans* mutants are avirulent. Cell 90:939–949. doi:10.1016/s0092-8674(00)80358-x. 9298905

[8] Braun BR, Johnson AD 1997 Control of filament formation in *Candida albicans* by the transcriptional repressor *TUP1*. Science 277:105–109. doi:10.1126/science.277.5322.105. 9204892

[9] Braun BR, Head WS, Wang MX, Johnson AD 2000 Identification and characterization of *TUP1*-regulated genes in *Candida albicans*. Genetics 156:31–44. 10978273 10.1093/genetics/156.1.31 PMC1461230

[10] Saville SP, Lazzell AL, Monteagudo C, Lopez-Ribot JL 2003 Engineered control of cell morphology *in vivo* reveals distinct roles for yeast and filamentous forms of *Candida albicans* during infection. Eukaryot Cell 2:1053–1060. doi:10.1128/ec.2.5.1053-1060.2003. 14555488 PMC219382

[11] Zheng X, Wang Y, Wang Y 2004 Hgc1, a novel hypha-specific G1 cyclin-related protein regulates *Candida albicans* hyphal morphogenesis. EMBO J 23:1845–1856. doi:10.1038/sj.emboj.7600195. 15071502 PMC394249

[12] Carlisle PL, Banerjee M, Lazzell A, Monteagudo C, Lopez-Ribot JL, Kadosh D 2009 Expression levels of a filament-specific transcriptional regulator are sufficient to determine *Candida albicans* morphology and virulence. Proc Natl Acad Sci U S A 106:599–604. doi:10.1073/pnas.0804061106. 19116272 PMC2626749

[13] Moran GP, Sullivan DJ, Coleman DC 2002 Emergence of non-*Candida albicans Candida* species as pathogens, p 37–54. *In* Calderone RA (ed), *Candida* and candidiasis. ASM Press, Washington, DC.

[14] Priest SJ, Lorenz MC 2015 Characterization of virulence-related phenotypes in *Candida* species of the CUG clade. Eukaryot Cell 14:931–940. doi:10.1128/EC.00062-15. 26150417 PMC4551586

[15] Lackey E, Vipulanandan G, Childers DS, Kadosh D 2013 Comparative evolution of morphological regulatory functions in *Candida* species. Eukaryot Cell 12:1356–1368. doi:10.1128/EC.00164-13. 23913541 PMC3811340

[16] Zhang Q, Tao L, Guan G, Yue H, Liang W, Cao C, Dai Y, Huang G 2016 Regulation of filamentation in the human fungal pathogen *Candida tropicalis*. Mol Microbiol 99:528–545. doi:10.1111/mmi.13247. 26466925

[17] Brancaccio P, Lippi G, Maffulli N 2010 Biochemical markers of muscular damage. Clin Chem Lab Med 48:757–767. doi:10.1515/CCLM.2010.179. 20518645

[18] Carlisle PL, Kadosh D 2013 A genome-wide transcriptional analysis of morphology determination in *Candida albicans*. Mol Biol Cell 24:246–260. doi:10.1091/mbc.E12-01-0065. 23242994 PMC3564527

[19] Sinha I, Wang YM, Philp R, Li CR, Yap WH, Wang Y 2007 Cyclin-dependent kinases control septin phosphorylation in *Candida albicans* hyphal development. Dev Cell 13:421–432. doi:10.1016/j.devcel.2007.06.011. 17765684

[20] Silva S, Henriques M, Oliveira R, Azeredo J, Malic S, Hooper SJ, Williams DW 2009 Characterization of *Candida parapsilosis* infection of an in vitro reconstituted human oral epithelium. Eur J Oral Sci 117:669–675. doi:10.1111/j.1600-0722.2009.00677.x. 20121929

[21] Toth R, Cabral V, Thuer E, Bohner F, Nemeth T, Papp C, Nimrichter L, Molnar G, Vagvolgyi C, Gabaldon T, Nosanchuk JD, Gacser A 2018 Investigation of *Candida parapsilosis* virulence regulatory factors during host-pathogen interaction. Sci Rep 8:1346. doi:10.1038/s41598-018-19453-4. 29358719 PMC5777994

[22] Yu S, Li W, Liu X, Che J, Wu Y, Lu J 2016 Distinct expression levels of *ALS*, *LIP*, and *SAP* genes in *Candida tropicalis* with diverse virulent activities. Front Microbiol 7:1175. doi:10.3389/fmicb.2016.01175. 27524980 PMC4965447

[23] Silva S, Hooper SJ, Henriques M, Oliveira R, Azeredo J, Williams DW 2011 The role of secreted aspartyl proteinases in *Candida tropicalis* invasion and damage of oral mucosa. Clin Microbiol Infect 17:264–272. doi:10.1111/j.1469-0691.2010.03248.x. 20456460

[24] Jiang C, Li Z, Zhang L, Tian Y, Dong D, Peng Y 2016 Significance of hyphae formation in virulence of *Candida tropicalis* and transcriptomic analysis of hyphal cells. Microbiol Res 192:65–72. doi:10.1016/j.micres.2016.06.003. 27664724

[25] Chen YV, Rosli R, Fong SH, Sidik SM, Pei CP 2012 Histopathological characteristics of experimental *Candida tropicalis* induced acute systemic candidiasis in BALB/c mice. Int J Zool Res 8:12–22. doi:10.3923/ijzr.2012.12.22.

