# Supplementary figures and images for: Filamentation Is Associated with Reduced Pathogenicity of Multiple Non-albicans Candida Species
Source: mSphere. 2019 Oct 16;4(5):e00656-19. doi: 10.1128/mSphere.00656-19 (PMC6796982; doi:10.1128/mSphere.00656-19)

A

*C. parapsilosis*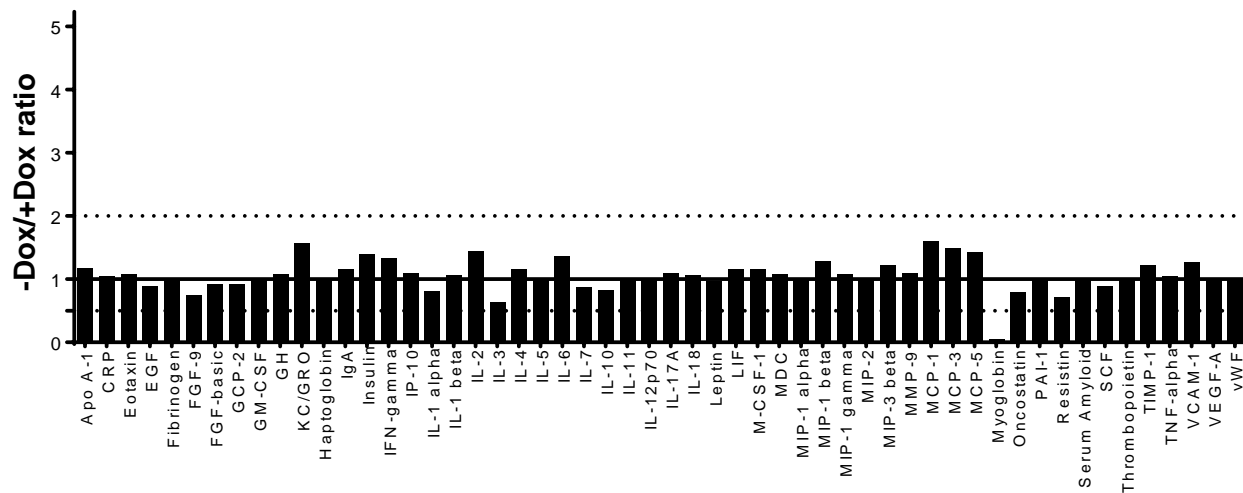

B

*C. tropicalis*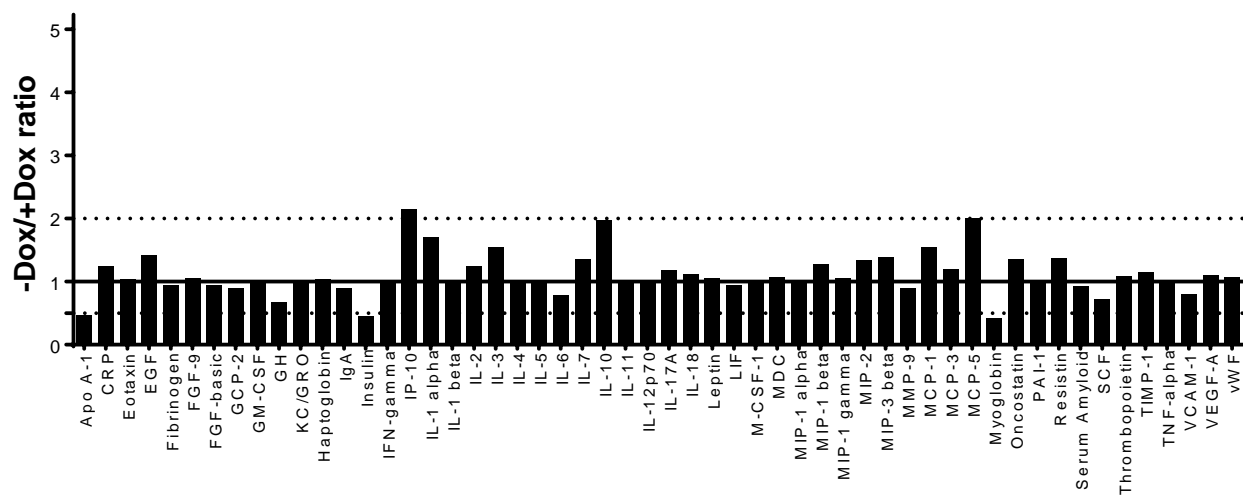

Figure S1

Supplement: FIG S1 [file mSphere.00656-19-sf001.pdf]

**A*****C. parapsilosis*****+ Dox****- Dox****WT**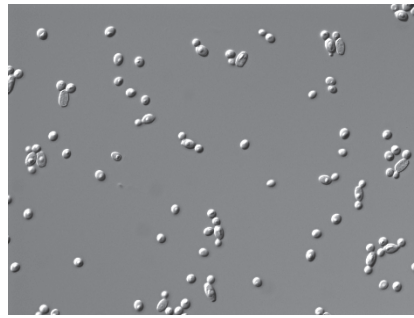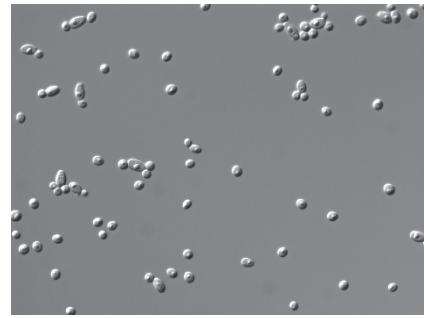***tetO-CpUME6***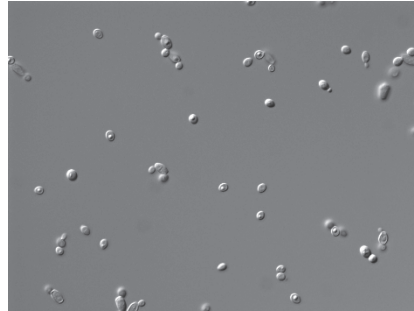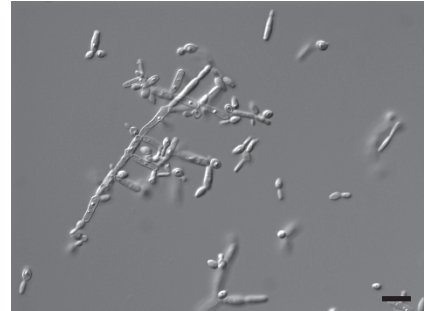**B*****C. tropicalis*****+ Dox****- Dox****WT**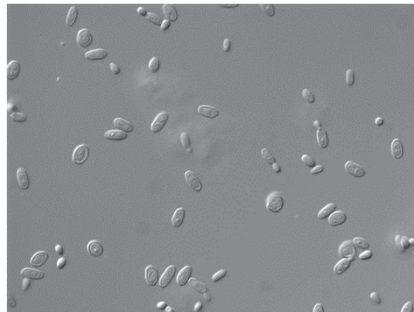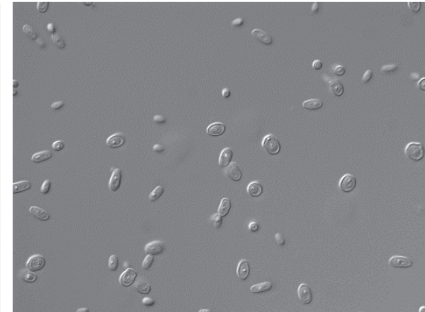***tetO-CtUME6***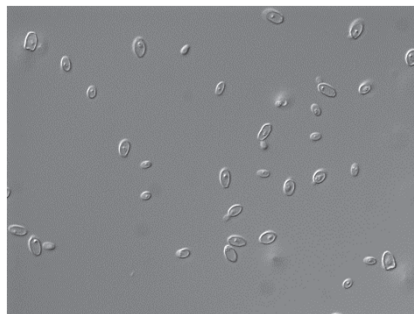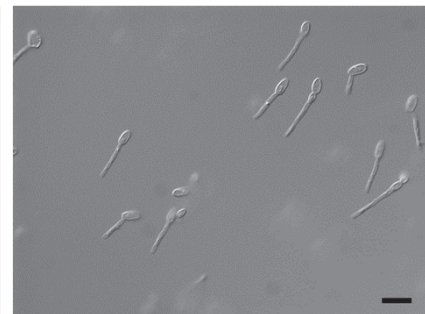**Figure S2**

Supplement: FIG S2 [file mSphere.00656-19-sf002.pdf]
