# Supplementary material for: Filamentation Is Associated with Reduced Pathogenicity of Multiple Non-albicans Candida Species
Source: mSphere. 2019 Oct 16;4(5):e00656-19. doi: 10.1128/mSphere.00656-19 (PMC6796982; doi:10.1128/mSphere.00656-19)
Supplement: TEXT S1 [file mSphere.00656-19-s0001.docx]

**Text S1: Supplemental Materials and Methods**

**Strains and growth conditions**

*C. parapsilosis* wild-type (ELY70) and *tetO-CpUME6* (ELY194) strains as well as *C. tropicalis* wild-type (ELY5), *nrg1*Δ/Δ (ELY145) and *tetO-CtUME6* (ELY184) strains have been previously described ([1](#_ENREF_1)). *C. tropicalis* strains MBY316 (*hgc1*Δ/Δ) and MBY320 (WT) were used for the pathogenicity experiment in Figure 1F. These strains were generated from strains *hgc1*/*hgc1* and G1374h ([2](#_ENREF_2)), respectively, by the integration of a single *C. tropicalis* *HIS1* marker (generated using primers MBO208 and MBO209 (Table S3)) at the *C. tropicalis* *his1*/*his1* locus. Standard growth conditions for all strains were in yeast extract-peptone-dextrose (YEPD) at 30°C ([3](#_ENREF_3)). For animal experiments, strains were grown in the presence and absence of doxycycline (Dox) as described previously ([4](#_ENREF_4)). For RNA preparation and cell fixing, strains were initially grown overnight at 30°C in 5 mL of YEPD in the presence (*tetO-CpUME6* and *tetO-CtUME6* strains) or absence (*Cp*WT and *Ct*WT strains) of 20 μg/mL Dox. Saturated overnight cultures were washed twice in ddH_2_O and resuspended in 5 mL ddH_2_O. Next, 10 μL of this cell suspension was diluted in 10 mL of ddH_2_O. For *C. parapsilosis* strains, either 1 mL or 2 mL of this diluted culture was used to inoculate 50 mL of synthetic complete (SC) medium in the presence and absence of 100 ng/mL Dox and cells were grown at 30°C for 36 hours prior to harvesting 1 mL for fixing and 5 mL for RNA preparation. For *C. tropicalis* strains, either 1 mL or 2 mL of the diluted culture was used to inoculate 50 mL of YEPD + 50% fetal bovine serum (FBS) (Atlanta Biologicals) in the presence and absence of 100 ng/mL Dox and cells were grown at 30°C for 24 hours prior to harvesting 1 mL for fixing and 5 mL for RNA preparation. We have previously observed that different growth conditions are required for optimal filamentation of *tetO-CpUME6* and *tetO-CtUME6* strains in the absence of Dox (1).

**Pathogenicity assays**

Strains were assessed for pathogenicity in the presence and absence of Dox using the mouse model of systemic candidiasis as described previously ([4](#_ENREF_4)). At each sacrifice point, organs were harvested for both histological and fungal burden analysis as previously described ([4](#_ENREF_4), [5](#_ENREF_5)). The Mann-Whitney test was used to determine statistically significant differences in organ fungal burden. This analysis was carried out using Prism by Graphpad Software, Inc. (San Diego, CA).

**Immune profiling**

Mice on drinking water containing 5% sucrose in the presence and absence of 2 mg/mL Dox were inoculated with either 4.2 x 10^6^ CFUs of the *tetO-CpUME6* strain or 2.1 x 10^5^ CFUs of the *tetO-CtUME6* strain and sacrificed 1 day post-infection. Pooled kidney homogenates from each group were used to assess levels of a variety of cytokines, chemokines and other immune markers by a Luminex^®^-based Multi-Analyte Profile (MAP) technology platform (Rules Based Medicine, Austin, TX) as described previously ([6](#_ENREF_6)).

**RNA preparation**

Total RNA was prepared using the hot acid phenol method, as described previously ([7](#_ENREF_7)).

**Transcriptional Profiling**

cDNA libraries for RNA-seq analysis were prepared from total RNA samples using an Illumina TruSeq stranded mRNA-seq kit. RNA sequencing was performed in biological duplicate using an Illumina HiSeq 2000 machine at the Greehey Children’s Cancer Research Institute Genome Sequencing Facility (University of Texas Health Science Center at San Antonio) to obtain 100 bp paired-end reads. Reads were trimmed for adaptor sequences, masked for low-quality/low complexity sequences and mapped to genome assemblies for *C. tropicalis* MYA3404 and *C. parapsilosis* CDC317 obtained from the *Candida* Genome Database ([www.candidagenome.org](http://www.candidagenome.org)) using TopHat v.2.0.8b software. Gene count measurements were obtained using HTSeq-count and differential expression analysis was carried out using the DESeq package. Datasets 1-4 contain genes that show a ≥ 2-fold change in expression in the absence vs. presence of Dox for the *tetO-CpUME6* or *tetO-CtUME6* strains and p_adj_ ≤ 0.05; expression data for each gene in *Cp*WT or *Ct*WT strains is also shown. Genes that also show a corresponding ≥ 2-fold change in expression (p_adj_ ≤ 0.05) in the absence vs. presence of Dox in respective wild-type strains are not included in these datasets. Gene annotation was obtained from the *Candida* Genome Database ([www.candidagenome.org](http://www.candidagenome.org)) and *Candida* Gene Order Browser (<http://cgob.ucd.ie>). Please note that *C. tropicalis* gene annotation is based on that of *C. albicans* gene orthologs. Gene ontology (GO) analysis was carried out using the GO Slim Mapper and GO Term Finder tools (default settings) available at the *Candida* Genome Database. For genes which have multiple orthologs in different species, the species priority order for determining the number of genes reported in Venn diagrams was *C. tropicalis*, *C. parapsilosis*, *C. albicans*.

**References**

1. Lackey E, Vipulanandan G, Childers DS, Kadosh D. 2013. Comparative evolution of morphological regulatory functions in *Candida* species. Eukaryot Cell 12:1356-68.

2. Zhang Q, Tao L, Guan G, Yue H, Liang W, Cao C, Dai Y, Huang G. 2016. Regulation of filamentation in the human fungal pathogen *Candida tropicalis*. Mol Microbiol 99:528-45.

3. Guthrie C, Fink GR. 1991. Guide to yeast genetics and molecular biology. Academic Press, San Diego.

4. Carlisle PL, Banerjee M, Lazzell A, Monteagudo C, Lopez-Ribot JL, Kadosh D. 2009. Expression levels of a filament-specific transcriptional regulator are sufficient to determine *Candida albicans* morphology and virulence. Proc Natl Acad Sci U S A 106:599-604.

5. Saville SP, Lazzell AL, Monteagudo C, Lopez-Ribot JL. 2003. Engineered control of cell morphology *in vivo* reveals distinct roles for yeast and filamentous forms of *Candida albicans* during infection. Eukaryot Cell 2:1053-60.

6. Chaturvedi AK, Lazzell AL, Saville SP, Wormley FL, Jr., Monteagudo C, Lopez-Ribot JL. 2011. Validation of the tetracycline regulatable gene expression system for the study of the pathogenesis of infectious disease. PLoS One 6:e20449.

7. Ausubel FM, Brent R, Kingston RE, Moore DD, Seidman JG, Smith JA, Struhl K (ed). 1992. Current protocols in molecular biology. Greene Publishing Associates and Wiley-Interscience, New York.
