# Supplementary material for: Filamentation Is Associated with Reduced Pathogenicity of Multiple Non-albicans Candida Species
Source: mSphere. 2019 Oct 16;4(5):e00656-19. doi: 10.1128/mSphere.00656-19 (PMC6796982; doi:10.1128/mSphere.00656-19)
Supplement: TABLE S2 [file mSphere.00656-19-st002.docx]

**Table S2.** Number of genes showing altered expression in the absence vs. presence of doxycycline in *Candida tropicalis tetO-CtUME6* and wild-type strains.

|  | **Fold change (-Dox/+Dox)*** | | |
| --- | --- | --- | --- |
|  | **≥ 2-fold** | **≥ 4-fold** | **≥ 10-fold** |
| **# of genes up-regulated in *tetO-CtUME6*** | 1,052 | 389 | 147 |
| **# of genes up-regulated in *Ct* WT** | 15 | 1 | 0 |
|  | **≥ 2-fold** | **≥ 4-fold** | **≥ 10-fold** |
| **# of genes down-regulated in *tetO-CtUME6*** | 1,140 | 406 | 152 |
| **# of genes down-regulated in *Ct* WT** | 18 | 2 | 0 |

* Fold changes are based on mean gene expression values from cells grown in the absence vs. presence of 100 ng/mL doxycycline (Dox) from two independent RNA-seq experiments (n=2, p_adj_ ≤ 0.05)

*Ct* = *Candida tropicalis*. WT = wild-type.
