# Supplementary material for: Filamentation Is Associated with Reduced Pathogenicity of Multiple Non-albicans Candida Species
Source: mSphere. 2019 Oct 16;4(5):e00656-19. doi: 10.1128/mSphere.00656-19 (PMC6796982; doi:10.1128/mSphere.00656-19)
Supplement: TABLE S3 [file mSphere.00656-19-st003.docx]

| **Table S3 Primers used in this study** | | | | | | |
| --- | --- | --- | --- | --- | --- | --- |
| # | Primer Name | Sequence | | Description | | |
| 1 | MBO208 | | GGGTTTGATAGGTACATGGACG | | *C. tropicalis HIS1* forward primer |  |
| 2 | MBO209 | CGGTGATACTGTGCAATTTGCCG | | *C. tropicalis HIS1* reverse primer | | |
